# Ketogenic Diet as Preferred Treatment of FIRES

**DOI:** 10.15844/pedneurbriefs-29-1-2

**Published:** 2015-01

**Authors:** John J. Millichap, J. Gordon Millichap

**Affiliations:** 1 Division of Neurology, Ann & Robert H. Lurie Children's Hospital of Chicago, Chicago, IL; 2 Departments of Pediatrics and Neurology, Northwestern University Feinberg School of Medicine, Chicago, IL

**Keywords:** Epilepsy, Ketogenic diet, Encephalitis

## Abstract

Investigators from the University of Alabama, Birmingham, AL, and University of Michigan, Ann Arbor, MI, report 2 children who presented with FIRES and prolonged AED-resistant status epilepticus.

Investigators from the University of Alabama, Birmingham, AL, and University of Michigan, Ann Arbor, MI, report 2 children who presented with FIRES and prolonged AED-resistant status epilepticus. Patient 1 tested negative for infectious, metabolic, genetic, and autoimmune etiologies. The EEG showed burst suppression that transitioned to periodic epileptiform discharges, and a brain MRI showed changes consistent with bilateral mesial sclerosis. Patient 2 EEG showed burst suppression followed by temporal seizure foci, and a normal MRI. Infectious, rheumatologic, and autoimmune investigations were negative. Seizures were treated successfully with the ketogenic diet (4:1 ratio) in the acute and chronic stages of the encephalopathy. Both children were maintained on the diet along with AEDs for several months (20 and 18-month follow-up). Their cognitive outcome was moderately impaired (IQ 71 and 62) and they returned to school with some academic accommodations and an individualized education plan (IEP). Early treatment with the ketogenic diet is recommended as the preferred treatment for acute and long-term management of FIRES. [1]

COMMENTARY. Febrile infection-related epilepsy syndrome (FIRES) is a pseudo-encephalitic epileptic encephalopathy that presents with drug-resistant status epilepticus in previously healthy school-aged children [2]. The underlying pathophysiology is usually undetermined but may be immune-mediated. Treatment with anticonvulsants (e.g. phenobarbital, topiramate), immunoglobulin, steroids, and plasmapheresis) has limited success [3], and the ketogenic diet is more effective [1]. Numerous theories are advanced for the mechanism of the ketogenic diet but none is proven. The list of proposed mechanisms [4] begins with an anesthetic effect of ketone bodies and is followed by changes in electrolyte, pH, and water balance. Studies in the Clinic and in laboratory animals found the anticonvulsant effect of the diet is independent of a respiratory or metabolic acidosis, unrelated to diuresis, and correlated with a negative systemic balance of sodium and potassium, effects similar to those of acetazolamide [5]. Subsequent theories include an increase in cerebral energy reserves with no observed change in brain pH, water content or electrolytes [6], the inhibitory actions of polyunsaturated fatty acids (PUFAs), elevated GABA and decreased glutamate levels in CSF, and effects on noradrenergic system, benzodiazepine receptor, glycolysis and on activated potassium channels (K-ATP).

Compared to antiepileptic drugs, the safety of the diet is generally regarded as high. However, the recent increased popularity of the diet has drawn attention to the long-term but reversible effects on blood lipids and arterial function. The use of diets with less fat, such as the modified Atkins diet and low glycemic index treatment, is proposed as a safer alternative. Discontinuation of the diet after 6 months to 2 years if seizures are controlled should be considered, but a hasty removal of an effective therapy based on preliminary adverse data should be discouraged.
